# Multilevel Genome Typing Describes Short- and Long-Term Vibrio cholerae Molecular Epidemiology

**DOI:** 10.1128/mSystems.00134-21

**Published:** 2021-08-24

**Authors:** Liam Cheney, Michael Payne, Sandeep Kaur, Ruiting Lan

**Affiliations:** a School of Biotechnology and Biomolecular Science, University of New South Wales, Sydney, New South Wales, Australia; Pacific Northwest National Laboratory

**Keywords:** classification, epidemiology, multilevel genome typing, outbreak, phylogenetic relationships, seventh pandemic, standardization, transmission, typing, *Vibrio cholerae*

## Abstract

Since 1817, cholera, caused by Vibrio cholerae, has been characterized by seven distinct pandemics. The ongoing seventh pandemic (7P) began in 1961. In this study, we developed a Multilevel Genome Typing (MGT) tool for classifying the V. cholerae species with a focus on the 7P. MGT is based on multilocus sequence typing (MLST), but the concept has been expanded to include a series of MLST schemes that compare population structure from broad to fine resolutions. The V. cholerae MGT consists of eight levels, with the lowest, MGT1, composed of 7 loci and the highest, MGT8, consisting of the 7P core genome (3,759 loci). We used MGT to analyze 5,771 V. cholerae genomes. The genetic relationships revealed by lower MGT levels recapitulated previous findings of large-scale 7P transmission across the globe. Furthermore, the higher MGT levels provided an increased discriminatory power to differentiate subgroups within a national outbreak. Additionally, we demonstrated the usefulness of MGT for non-7P classification. In a large non-7P MGT1 type, MGT2 and MGT3 described continental and regional distributions, respectively. Finally, MGT described trends of 7P in virulence, and MGT2 to MGT3 sequence types (STs) grouped isolates of the same *ctxB*, *tcpA*, and *ctxB*-*tcpA* genotypes and characterized their trends over the pandemic. MGT offers a range of resolutions for typing V. cholerae. The MGT nomenclature is stable, transferable, and directly comparable between investigations. The MGT database (https://mgtdb.unsw.edu.au/) can accept and process newly submitted samples. MGT allows tracking of existing and new isolates and will be useful for understanding future spread of cholera.

**IMPORTANCE** In 2017, the World Health Organization launched the “Ending Cholera” initiative to reduce cholera-related deaths by 90% by 2030. This strategy emphasized the importance of the speed and accessibility of newer technologies to contain outbreaks. Here, we present a new tool named Multilevel Genome Typing (MGT), which classifies isolates of the cholera-causing agent, Vibrio cholerae. MGT is a freely available online database that groups genetically similar V. cholerae isolates to quickly indicate the origins of outbreaks. We validated the MGT database retrospectively in an outbreak setting, showcasing rapid confirmation of the Nepalese origins for the 2010 Haiti outbreak. In the past 5 years, thousands of V. cholerae genomes have been submitted to the NCBI database, which underscores the importance of and need for proper genome data classification for cholera epidemiology. The V. cholerae MGT database can assist in early decision making that directly impacts controlling both the local and global spread of cholera.

## INTRODUCTION

Cholera, caused by Vibrio cholerae, is an acute diarrheal disease transmitted through fecal contamination of water. Modern history (1816 to present) records seven cholera pandemics. The first six originated from the Ganges Delta, and the current, seventh pandemic (7P) arose in Indonesia (1). V. cholerae is divided into more than 200 serogroups by O antigen serogrouping (2). Of these, the O1 serogroup is responsible for all seven pandemics and is further divided into the classical and El Tor biotypes (2). The ongoing seventh pandemic began in 1961 and has spread across the globe in a series of three waves (3). The 7P is distinguished from the previous sixth pandemic by a shift in etiological agent from O1 classical to O1 El Tor. The seventh pandemic clone can be further divided based on allelic variation in the major virulence-associated genes *ctxB* and *tcpA*. The former is within the integrated cholera toxin (CTX) prophage, while the latter is located in the V. cholerae pathogenicity island (VPI) (4, 5). The capability of MGT to describe groups of isolates defined by allelic variation of *ctxB* and *tcpA* genotypes was examined.

Whole-genome sequencing has provided unprecedented resolution to distinguish isolates within the 7P. To date, phylogenies based on thousands of single-nucleotide polymorphism (SNP) differences from genome sequences have been used to investigate both large-scale 7P transmission throughout Asia, Africa, and South America, and the sources of the recent Haiti and Yemen outbreaks (3, 6–9). While the use of SNP-based phylogenetics resolves the genetic relationships of isolates, it lacks the flexibility to include additional data without reanalysis of the previous data. Allele-based typing, such as multilocus sequence typing (MLST), provides an alternative approach that can be standardized and is transferable between investigations (10). MLST compares alleles in a set of shared loci, and a sequence type (ST) is assigned to represent a unique combination of alleles for those loci (10). Both the classic seven-gene MLST scheme and larger core genome MLST (cgMLST) schemes have been developed for V. cholerae (11, 12). The seven-gene MLST scheme defines nearly the entire 7P as a single type, ST69, while cgMLST divides the 7P into hundreds of STs with little to no population structure defined by each ST (12). Additionally, clustering of cgMLST data using suitable allelic thresholds can group cgMLST STs with informative divisions. Liang et al. (12) found that 133 allelic differences clustered isolates into sublineages that are nearly identical to traditional MLST types, and seven allelic differences can group isolates that cause outbreaks. However, clusters defined using allelic distance may not be stable and are subject to merging when newly analyzed isolates satisfy the allelic difference threshold for multiple clusters.

Multilevel Genome Typing (MGT) alleviates the limitations of single-sized MLST schemes by expanding the MLST concept to a series of schemes of increasing size (13). This range of schemes offers flexibility when examining population structure by providing multiple levels of resolution. In MGT, each analyzed isolate is assigned multiple STs, one at each MGT level. The MGT nomenclature reflects genetic population structure and should provide a stable and globally standardized basis for small- and large-scale genomic epidemiology. In this study, we developed an eight-level MGT scheme to provide a range of resolutions for genomic typing of V. cholerae isolates. We utilize the various levels of the MGT scheme to explore both large- and small-scale 7P epidemiology. The lower resolution MGT levels (e.g., MGT2 and MGT3) described the large-scale epidemiology over continents and were used to explore the 7P population structure, while the higher resolution levels (e.g., MGT5) characterized diversity within a national outbreak. Finally, we used MGT to classify non-7P isolates, and MGT2 and MGT3 described the continental and regional distributions, respectively, of the largest non-7P MGT1 type.

## RESULTS

### MGT design—assigning core loci to the eight MGT levels.

**FIG 1:**
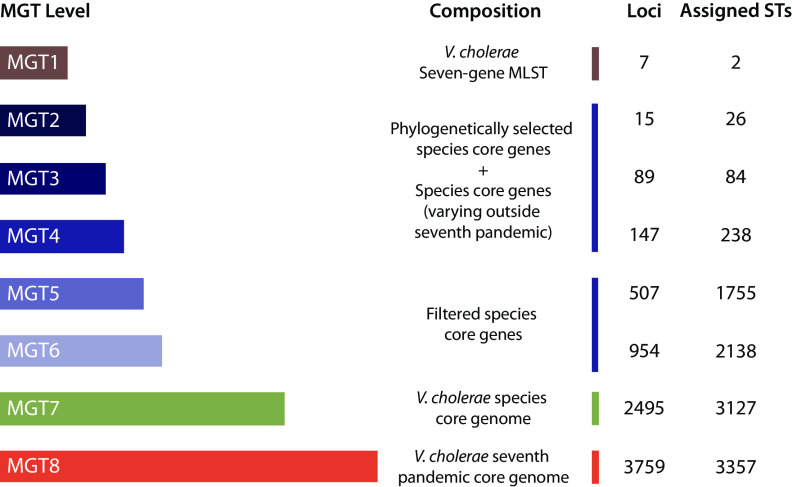
Vibrio cholerae Multilevel Genome Typing (MGT). The seventh pandemic (7P) MGT consists of eight multilocus sequence typing (MLST) schemes referred to as levels. Colored bars represent each level and vary in width depending on the number of loci. The method of loci selection, number of loci, and number of assigned sequence types (STs) after MGT analysis of 4,770 7P isolates are labeled next to each level.

Here, we created a novel genomic classification system for typing V. cholerae isolates. MGT expands the traditional MLST concept to include a series of MLST schemes (called levels) with increasing loci numbers. The V. cholerae MGT scheme consists of eight levels, MGT1 to MGT8 (Fig. 1). MGT1 was a previously established seven-gene MLST scheme (11). For MGT7 and MGT8, two core genomes were defined, a V. cholerae species core with 2,495 genes and a V. cholerae 7P core with 3,759 core loci (2,854 genes and 905 intergenic regions [IGRs]) (see Text S1, “Supplementary Results 2.1,” and Fig. S2 in the supplemental material). The typing resolution of MGT8 was compared to that of SNP-based typing (Text S1, “Supplementary Results 2.2”). The distribution of pairwise distances between isolates defined by both methods was highly similar. For every SNP difference, there were 0.81 allele differences defined by MGT8 (Fig. S3).

10.1128/mSystems.00134-21.1 TEXT S1 Supplementary methods and results for Multilevel Genome Typing (MGT) design and application to V. cholerae isolates. Download Text S1, DOCX file, 0.09 MB. Copyright © 2021 Cheney et al. 2021 Cheney et al. https://creativecommons.org/licenses/by/4.0/ This content is distributed under the terms of the Creative Commons Attribution 4.0 International license.

10.1128/mSystems.00134-21.5 FIG S2 Definition of V. cholerae and V. cholerae seventh pandemic (7P) core loci. Selecting the BLAST nucleotide identity (BNI) thresholds for defining the V. cholerae species and 7P core genomes. (A to C) The highest nucleotide identity that did not substantially reduce the number of core loci was selected. The grey box and dotted line showed the BNI at which core loci began to plateau. (A) Defining species core genes; (B) defining 7P core genes; (C) defining 7P core intergenic regions. Download FIG S2, EPS file, 1.1 MB. Copyright © 2021 Cheney et al. 2021 Cheney et al. https://creativecommons.org/licenses/by/4.0/ This content is distributed under the terms of the Creative Commons Attribution 4.0 International license.

10.1128/mSystems.00134-21.6 FIG S3 Concordance of allele and single-nucleotide polymorphism (SNP) pairwise distances. Pairwise distances for 300 7P pandemic representatives were calculated based on MGT8 alleles and SNPs. (A) Distribution of pairwise allele and SNP distances. SNP distances were normalized to the size of the core genome, which was 77% of the N16916 reference genome. (B) The density of allele and SNP pairwise combinations were visualized. A theoretical 1:1 ratio of alleles to SNPs was overlaid (hashed line in grey). Higher-frequency pairwise distances are colored yellow, and less common distances are colored dark blue. Almost all pairwise combinations were placed below the grey hashed line to indicate that SNP typing was higher resolution than allele typing. Download FIG S3, EPS file, 2.6 MB. Copyright © 2021 Cheney et al. 2021 Cheney et al. https://creativecommons.org/licenses/by/4.0/ This content is distributed under the terms of the Creative Commons Attribution 4.0 International license.

**FIG 2:**
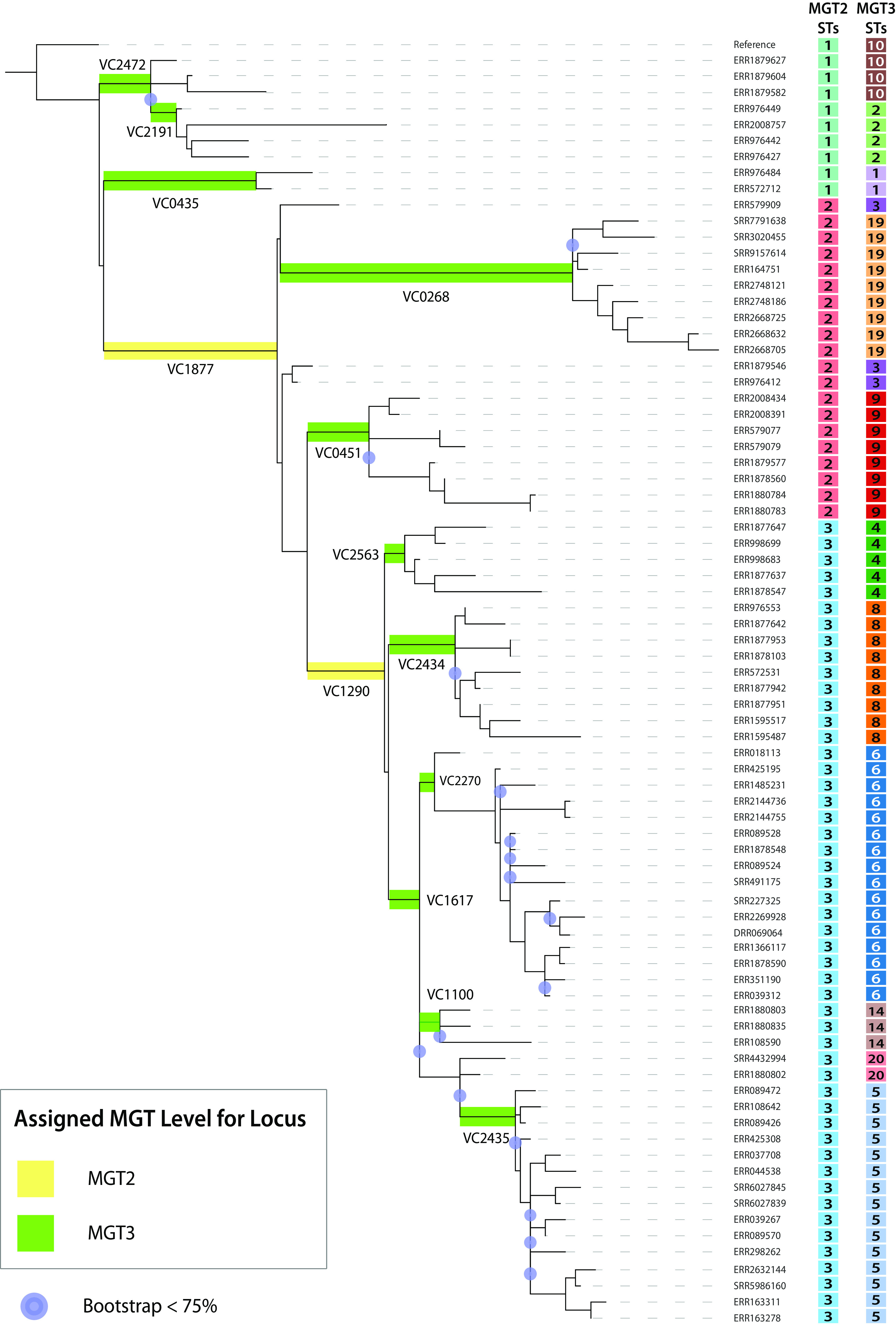
Selected MGT2 to MGT3 loci overlayed onto representative seventh pandemic phylogeny. A maximum-likelihood phylogeny was created from 80 representative isolates selected across the seventh pandemic (7P) using RAxML (42). Branches are highlighted with species core genes separating 7P clades. Mutations in genes within MGT2 and MGT3 are highlighted in yellow and green, respectively. The STs defined at MGT2 and MGT3 levels are aligned next to each isolate. Genetic relationships were derived from single-nucleotide polymorphism (SNP) differences in the 3,759 core 7P pandemic loci. The tree was rooted on the V. cholerae N16916 reference genome. Branches were bootstrapped 1,000 times, and branches showing less than 75% support are marked with a purple circle.

MGT2 to MGT6 contained subsets of the V. cholerae species core genome. The number of loci in these levels was calculated to reflect a range of time frames, so a single SNP would occur per given time frame at each level based on the V. cholerae N16916 whole-genome mutation rate (Text S1, “Supplementary Results 2.3”) (14). For MGT2 to MGT4, species core loci were selected so STs defined by these levels described clades of the 7P phylogenetic structure (Fig. 1) (detailed in “A standardized classification of the V. cholerae seventh pandemic with MGT2 to MGT4”). To achieve this, loci were selected that contained alleles unique to each clade of interest within the 7P. MGT2, MGT3, and MGT4 described 7P clades using 2, 11, and 50 loci, respectively (Fig. 2). Additionally, loci were identified that only differed outside the 7P, which allowed MGT2 to MGT4 to type non-7P isolates. MGT2, MGT3, and MGT4 were completed by adding 13, 78, and 97 of these non-7P variable loci, respectively (Fig. 1). The total numbers of loci for MGT2, MGT3, and MGT4 were 15, 89, and 147, respectively.

The remaining two middle levels, MGT5 and MGT6, had 507 and 954 loci, respectively (Fig. 1). The numbers of loci and the nucleotide lengths of these schemes was selected based on evolutionary time estimates. MGT5 and MGT6 schemes were sized so that, on average, one allelic change would occur every 5 years and every 1 year, respectively. These shorter timescales provided resolutions for shorter-term and smaller-scale epidemiology (Text S1, “Supplementary Results 2.4”) (13).

### A standardized classification of the V. cholerae seventh pandemic with MGT2 to MGT4.

Using the MGT levels, we analyzed V. cholerae 7P genomic data for 4,770 isolates (Data Set S1). As level increased, so did the number of STs that defined subsets of isolates within the 7P (Fig. 1). MGT1 classified the 7P into two MGT1 STs, ST69 and ST515, while MGT8 separated the 4,770 isolates into 3,357 STs. The eight MGT levels offered a range of resolutions for genomic typing that successively divided the 7P into smaller subgroups.

10.1128/mSystems.00134-21.2 DATA SET S1 Sequence types (STs) assigned at each Multilevel Genome Typing (MGT) level for all analyzed Vibrio cholerae isolates. Temporal, geographic, strain identification, and assembly quality metadata were added when available. Download Data Set S1, XLSX file, 0.6 MB. Copyright © 2021 Cheney et al. 2021 Cheney et al. https://creativecommons.org/licenses/by/4.0/ This content is distributed under the terms of the Creative Commons Attribution 4.0 International license.

MGT2 loci were selected so that MGT2 STs reflected the three waves of the 7P as previously described (3). MGT2 used 15 loci, of which only two differed among 7P isolates. These two loci, VC1290 and VC1877, had allelic variants that separated the isolates between waves. Wave one isolates had the N16961 reference allele for both loci. Wave two isolates had a variant in VC1877. Wave three isolates had a variant in both VC1877 and VC1290.

**FIG 3:**
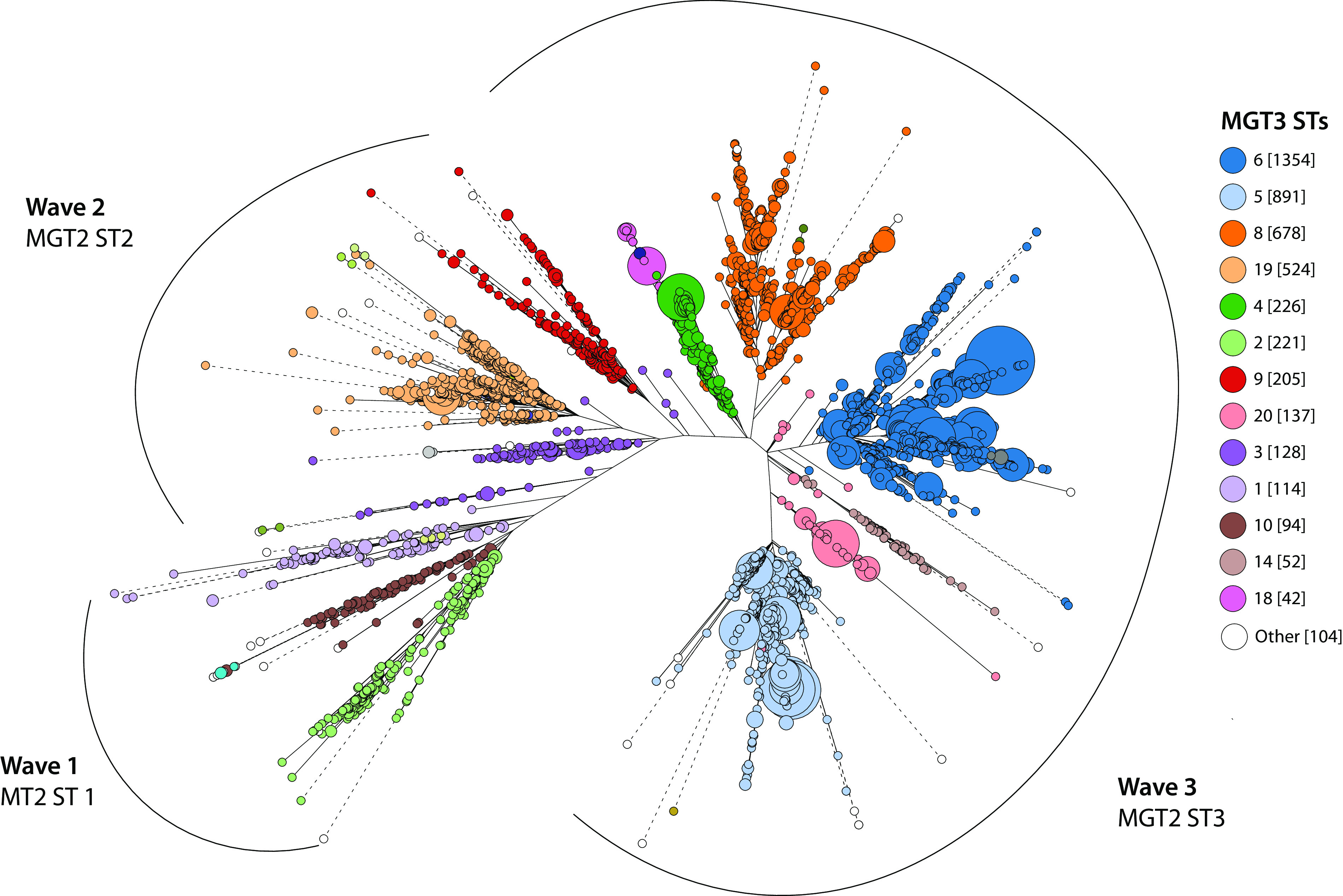
Seventh pandemic core genome phylogeny with MGT2 and MGT3 assignments. A 7P seventh pandemic (7P) core genome phylogeny based on MGT8 alleles was calculated and visualized with GrapeTree. Allelic distances of 4,770 7P isolates were used to generate a phylogeny using the rapid neighbor-joining algorithm (43). Nodes were MGT8 STs and node sizes were proportional to the number of isolates with the same MGT8 ST. Clades corresponding to 7P waves are indicated. Nodes are colored by MGT3 ST. The number of isolates per ST is shown in square brackets. MGT3 STs with less than 10 isolates were grouped into “Other” and are marked in white. Branches longer than 50 allelic differences were truncated and are represented as dashed lines.

The vast majority of isolates from waves one, two, and three were successfully assigned as MGT2 ST1 (445/452; 98%), MGT2 ST2 (885/897; 99%), and MGT2 ST3 (3,427/3,438; 99.6%), respectively (Fig. 3, outlined in black). MGT3 contained 11 loci that further divided MGT2 STs into 13 STs with more than 10 isolates each. MGT2 ST1 was separated into three MGT3 STs, MGT2 ST2 into three MGT3 STs, and MGT2 ST3 into seven MGT3 STs (Fig. 3, bubbles colored by MGT3 ST). MGT3 STs were either continent specific (8/13) or multicontinental (5/13) (*n* = 2,087 isolates with published metadata). Each wave had an MGT3 type that was spatially restricted to Asia, as follows: wave one, MGT3 ST7; wave two, MGT3 ST19; wave three, MGT3 ST5. A hallmark of the 7P (wave two) was the emergence and spread of the O139 serogroup throughout Asia (15). *In silico* screening of isolates for O-antigen type showed that MGT3 ST19 represented the O139 serogroup (524/550; 95%) (Text S1, “Supplementary Results 2.5”). This was supported by isolates previously confirmed as O139 by serological testing (15).

In addition to continent-specific STs, five intercontinental MGT3 STs were identified across the 7P. MGT3 ST6 arose during wave three and was the largest multicontinental ST of the 7P (1,354/4,770), and included isolates from Africa, Asia, Europe, and the Americas (Central and South) (Fig. 3, blue). Notably, MGT3 ST6 included the two largest cholera outbreaks in recent history, the 2010 Haiti outbreak and the 2016 Yemen outbreak (6). MGT4 STs described groups of isolates causing these outbreaks. The Haiti and Yemen outbreak clades were described by MGT4 ST11 and MGT4 ST12, respectively. Overall, the lower MGT2- and MGT3-level STs reflected phylogenetic structure. MGT2 recapitulated the three waves of the 7P, MGT3 identified continent specific and multicontinental lineages, and MGT4 identified recent major outbreaks.

### MGT application to non-7P isolates.

**FIG 4:**
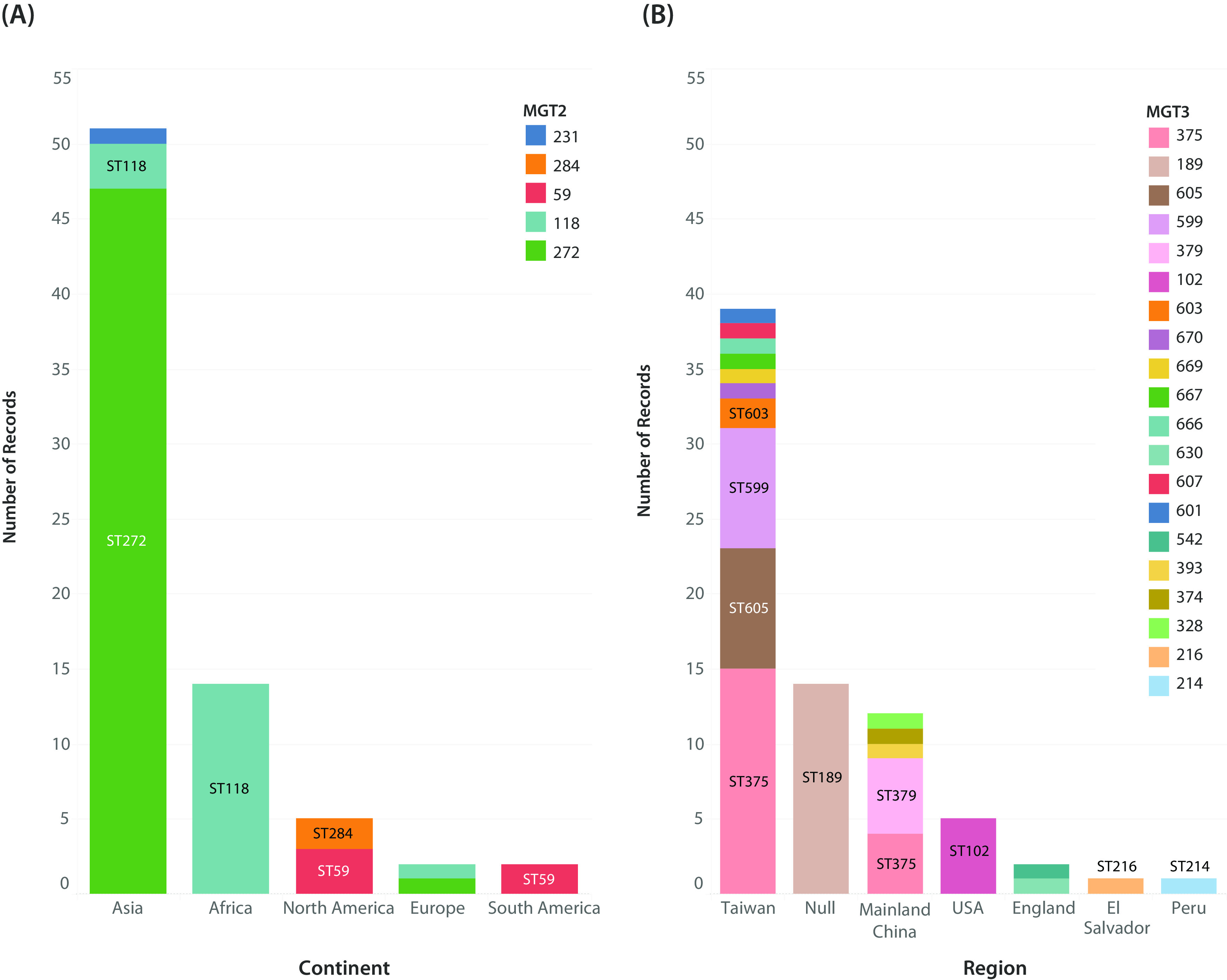
MGT STs divided non-seventh pandemic (7P) groups into continent and region. The largest non-7P MGT1 type, ST75, was divided by MGT2 and MGT3 (*n = 75*). MGT2 and MGT3 STs assigned to more than one isolate are overlaid. (A) MGT2 STs related to continent distribution. Isolates for each continent are colored by MGT2 type. (B) MGT3 STs related to region distribution. Isolates for each region are colored by MGT3 type. Visualized with Tableau v9.1 (41).

We verified that MGT levels 1 to 7 were able to classify non-7P isolates. MGT7, which is equivalent to the species cgMLST, was the highest level of resolution for these isolates, since MGT8 included loci specific to the 7P (Fig. 1A). MGT classified 97% (950/984) of publicly available non-7P isolates. Six MGT1 STs with more than 10 isolates each were divided into 32 MGT2 STs, and MGT7 assigned 80% of these (222/278) as singletons. MGT1 ST75 was the largest non-7P MGT1 ST (*n* = 150), and 75 of these isolates with region metadata were further investigated with higher MGT levels (Fig. 4). MGT2 separated MGT1 ST75 isolates into five STs that were related to continent of isolation (Fig. 4A). MGT2 ST272 isolates were predominantly Asian, MGT2 ST118 were African, and MGT2 ST59 and MGT2 ST284 were from the Americas. MGT3 further divided MGT1 ST75 into 20 STs (Fig. 4B). The majority of these STs were singletons (13/20), and 85% (5/7) of STs assigned to more than a single isolate were region specific. The American isolates were separated into MGT3 ST216, ST214, and ST102 for El Salvador, Peru, and the United States, respectively. A single MGT3 type, ST375, was detected in multiple regions of Asia, including mainland China and Taiwan. In summary, the MGT classified 97% of non-7P isolates. The largest MGT1 ST75 was divided by MGT2 and MGT3 and was related to continent and region of isolation, respectively.

### Application of MGT to tracing the origins of cholera in Haiti.

**FIG 5:**
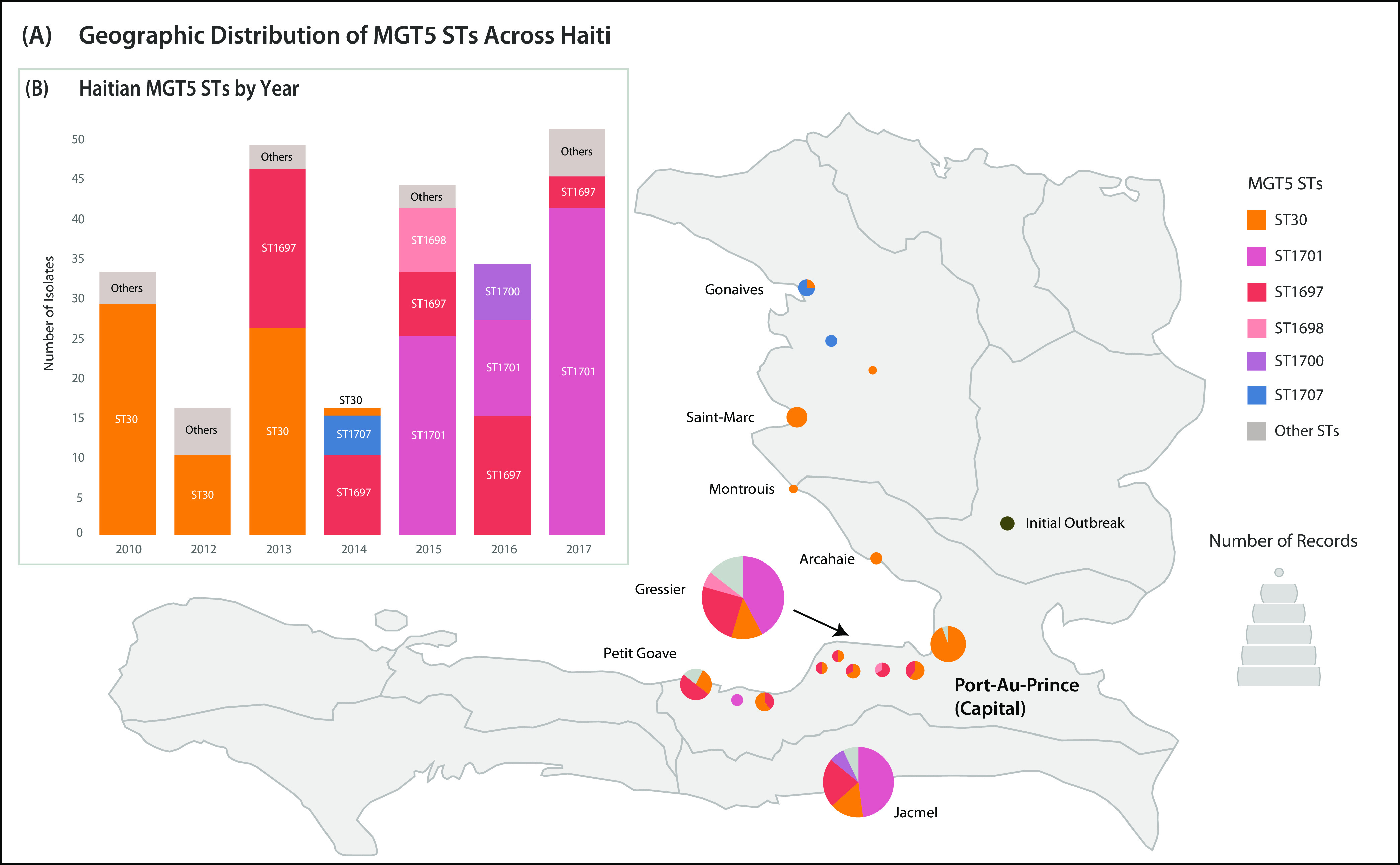
Geographic and temporal distribution of Haitian MGT5 STs. Isolates were collected between 2010 and 2017 from 11 locations. Coloring of STs between the bar chart and map is consistent. (A) The sizes of bubbles on the map are proportional to the numbers of isolates. Bubbles are divided based on MGT5 STs related to that location. The map was sourced from simplemaps (available at https://simplemaps.com/resources/svg-ht). (B) For each year, the number of isolates assigned to each MGT5 ST is shown. STs assigned to fewer than 10 isolates are collapsed into a single gray box. Visualized with Tableau v9.1 (41).

To demonstrate the utility of higher MGT resolutions, we examined 224 isolates from a recent Haitian outbreak (MGT4 ST11) (16). Initially, the population structure of Haiti outbreak was reconstructed phylogenetically (Fig. S4). This placed Indian and Nepalese isolates near the root, which agreed with previous reports that the Haiti outbreak originated from the Indian subcontinent (17). The Indian and Nepalese isolates were included in MGT4 ST11, showing that MGT4 ST11 was the source of the Haiti outbreak. At MGT5, MGT4 ST11 was divided into six STs with more than two isolates each, revealing dominant MGT5 STs circulating within Haiti (Fig. 5). MGT5 ST30 spread across 14 out of 17 sampled cities and was the most frequent MGT5 ST in the first 3 years sampled (2010 to 2013). In 2013, MGT5 ST30 was no longer dominant, and another MGT5 ST, ST1697, represented 42% (20/46) of samples. MGT5 ST1697 was the first MGT5 type to include isolates sourced from both environmental waters and clinical stool samples. After 2014, the original outbreak ST, MGT5 ST30, was no longer detected, and outbreaks consisted of MGT5 ST1697 and a newer ST, MGT5 ST1701 (Fig. 5A). A maximum-parsimony phylogeny of the Haitian outbreak isolates showed that MGT5 ST1697 and ST1701 isolates shared the most recent common ancestor with MGT5 ST30 outbreak isolates (Fig. S4). Based on this phylogeny, MGT5 ST1697 isolates evolved from MGT5 ST30, and MGT5 ST1701 subsequently evolved from MGT5 ST1697. This pattern suggests that the Haiti outbreak included three waves of outbreaks, with each evolving from and replacing its predecessor. In this outbreak case study, MGT5 resolution successfully separated populations within the Haiti outbreak. MGT5 identified three dominant STs, MGT5 ST30, ST1697, and ST1701, suggesting that this outbreak was a series of three epidemic waves.

10.1128/mSystems.00134-21.7 FIG S4 Complete phylogeny of Haitian outbreak clade. A maximum-parsimony phylogeny of 328 isolates with the same MGT4 ST as the Haiti outbreak clade was created. Isolates were distinguished based on 337 7P pandemic core SNPs. The tree was rooted using the reference wave one V. cholerae N16916 isolate. Blocks of color positioned to the right are related to the metadata categories MGT4 STs, MGT5 STs, country of origin, and year of isolation. Branches are colored based on bootstrap support values from 500 replicates. Bright green branches had a bootstrap support of 100%, and bright red branches had bootstrap supports of 0%. Leaf labels (accession numbers) were excluded. Scale bar represents a single SNP. Download FIG S4, EPS file, 1.6 MB. Copyright © 2021 Cheney et al. 2021 Cheney et al. https://creativecommons.org/licenses/by/4.0/ This content is distributed under the terms of the Creative Commons Attribution 4.0 International license.

### MGT typing can describe changing *ctxB-tcpA* genotypes.

**FIG 6:**
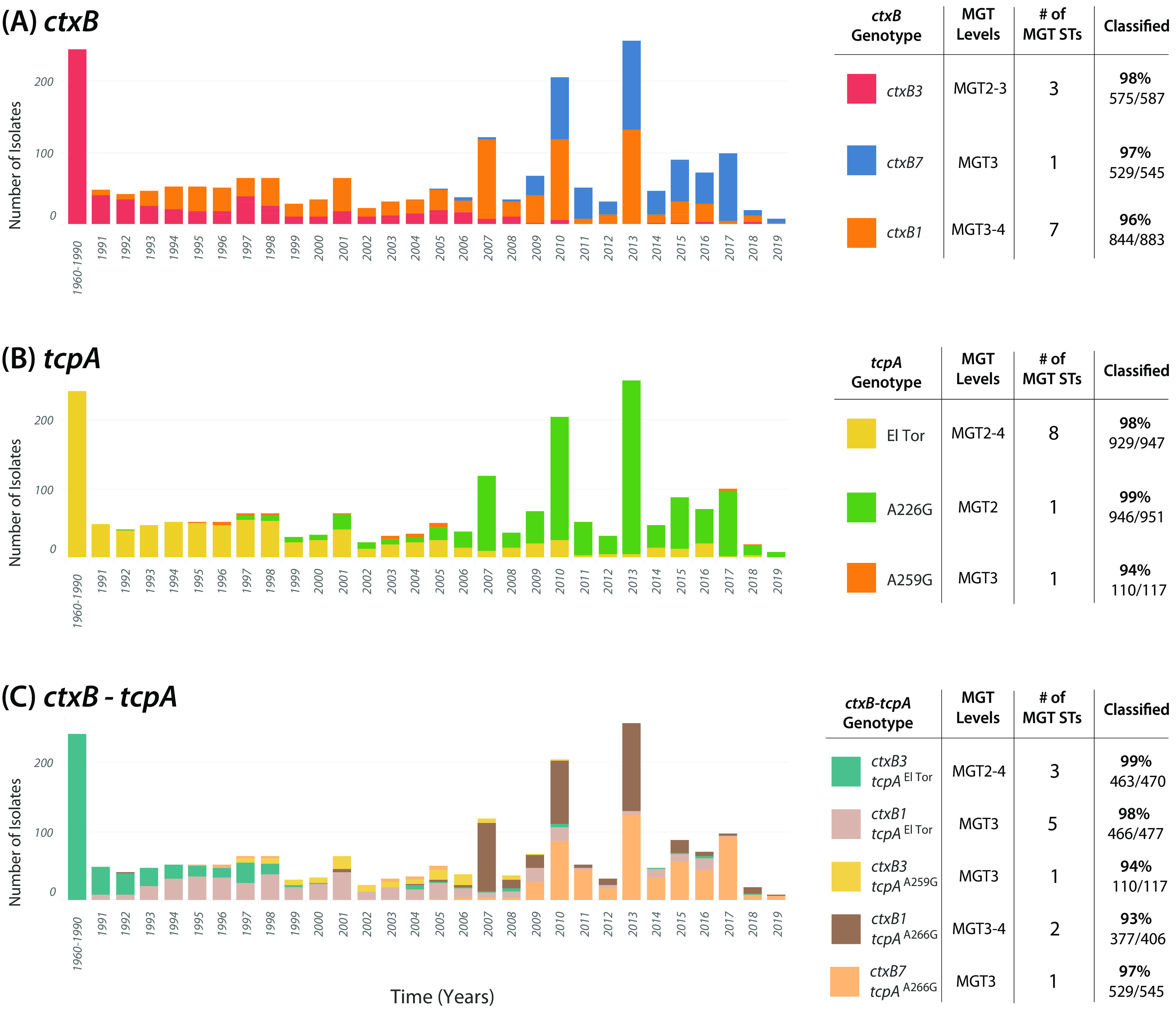
MGT STs characterized changing seventh pandemic *ctxB*, *tcpA*, and *ctxB*-*tcpA* genotypes. MGT STs from MGT2 to MGT4 described groups of isolates with the same *ctxB* (A), *tcpA* (B), *and ctxB-tcpA* (C) genotypes (*n* = 2,015). The frequency of isolates between 1961 and 2019 was as shown. The majority of genotypes were described with more than one ST. STs were mutually exclusive and were included if they contained more than 10 isolates. Isolates are colored by their variant for a marker gene. Graph was visualized using Tableau v9.1 (41).

Over the course of the 7P pandemic, virulence factors have been identified in the CTX and VPI regions (4, 5). Isolates with year metadata (*n* = 2,135) were screened *in silico*, and 51 unique virulence associated genes were predicted (Fig. S5). The *ctxB* and *tcpA* genes were present in more than 94% (2,015/2,135) of the isolates and included the following variants: *ctxB1*, *ctxB3*, and *ctxB7*, and *tcpA*^El Tor^, *tcpA*^A226G^, and *tcpA*^A259G^. When these two genes are, combined their 6 alleles produced 5 genotypes. The frequencies of *ctxB*, *tcpA*, and *ctxB*-*tcpA* genotypes were quantified from 1961 to 2019 (Fig. 6) (Data Set S2).

10.1128/mSystems.00134-21.3 DATA SET S2 *In silico* genotypes for seventh pandemic isolates with year metadata. Download Data Set S2, XLSX file, 0.2 MB. Copyright © 2021 Cheney et al. 2021 Cheney et al. https://creativecommons.org/licenses/by/4.0/ This content is distributed under the terms of the Creative Commons Attribution 4.0 International license.

10.1128/mSystems.00134-21.8 FIG S5 Frequencies of 46 virulence factor variants for the V. cholerae seventh pandemic. Variants for 46 V. cholerae virulence factors were quantified across a dataset of 7P pandemic isolates (*n* = 2,135). Variants within the virulence factor database were identified using Abricate (44, 45). Isolates with the same variant are colored per virulence factor. Arrows indicate *ctxB* and *tcpA* variants that were investigated in subsequent analysis. Download FIG S5, EPS file, 2.7 MB. Copyright © 2021 Cheney et al. 2021 Cheney et al. https://creativecommons.org/licenses/by/4.0/ This content is distributed under the terms of the Creative Commons Attribution 4.0 International license.

The ability for MGT nomenclature to describe groups of isolates with the same *ctxB*, *tcpA*, and *ctxB*-*tcpA* genotypes was examined. Description of different genotypes was investigated using STs from a single MGT level and STs from multiple MGT levels. Selecting STs from multiple levels described a higher percentage of isolates and used fewer STs (Text S1, “Supplementary Results 2.6”). A total of 33 STs from MGT2 to MGT4, on average, described 97% of isolates from each genotype (Fig. 6). For single-level description, MGT3 provided the best grouping of isolates of the same genotype (Fig. S6). A total of 36 STs from MGT3, on average, described 94% of isolates for each genotype (Fig. S7). Therefore, STs from multiple levels described a higher percentage of isolates and were used to further describe the frequencies of variants for each genotype. However, MGT provides the flexibility of using both.

10.1128/mSystems.00134-21.9 FIG S6 Number of isolates per MGT level described by virulence-specific MGT STs. The performance of the eight MGT levels to describe 2,015 isolates with the same virulence genotypes was visualized. STs were characterized as either genotype-specific, non-genotype-specific, or minor STs. Genotype-specific STs had more than 75% of isolates with the same genotype, and minor STs were assigned to fewer than 10 isolates. (A) Summary of the percentage of isolates described by genotype-specific STs. (B to D) For each *ctxB*, *tcpA*, and *ctxB*-*tcpA* genotype, the number of isolates in a ST that are either genotype specific, nonspecific, or minor. Isolates assigned as genotype-specific STs are shown in dark green, isolates assigned as non-genotype-specific STs are light green, and minor ST isolates are outlined in green. Download FIG S6, EPS file, 2.2 MB. Copyright © 2021 Cheney et al. 2021 Cheney et al. https://creativecommons.org/licenses/by/4.0/ This content is distributed under the terms of the Creative Commons Attribution 4.0 International license.

10.1128/mSystems.00134-21.10 FIG S7 Characterizing *ctxB*, *tcpA*, and *ctxB*-*tcpA* virulence genotypes by MGT3. A total of 36 MGT3 STs described groups of isolates with the same *ctxB* (A)*, tcpA* (B), and *ctxB-tcpA* (C) genotypes (*n* = 2,015). The frequency of isolates between 1961 and 2019 is shown. STs had more than 75% of isolates with a single virulence genotype and contained more than 10 isolates. Isolates are colored by their variant for a marker gene. Download FIG S7, EPS file, 1.2 MB. Copyright © 2021 Cheney et al. 2021 Cheney et al. https://creativecommons.org/licenses/by/4.0/ This content is distributed under the terms of the Creative Commons Attribution 4.0 International license.

The initial 7P genotype for each marker (*ctxB3*, *tcpA*^El Tor^, and *ctxB3*- *tcpA*^El Tor^) was the only one reported between 1961 and 1990. The first *ctxB* variant was *ctxB1* in 1991, and seven MGT STs described 96% of these isolates (Fig. 6A). After 2005, the majority of isolates carried either a *ctxB1* or a newer *ctxB7* allele (97% typed as MGT3 ST6). Ten STs from MGT2 to MGT4 separated isolates with three different *tcpA* variants (Fig. 6B). The earliest *tcpA* genotype, *tcpA*^El Tor^, was present in almost all sampled years (excluding 2017 to 2019). Almost all MGT2 ST3 isolates contained the *tcpA*^A226G^ variant (99%), which was the major genotype after 2006. A lower-frequency *tcpA*^A259G^ variant was carried in 117 isolates, and MGT3 ST19 typed 94% of these isolates.

Finally, isolates with the same combinations of *ctxB*-*tcpA* variants were described by MGT (Fig. 6C). Five genotypes were detected, and 12 MGT STs separated 97% of isolates with the same combination genotype. Before 1990, the earliest *ctxB*-*tcpA* genotype (*ctxB3*-*tcpA*^El Tor^) was most common, and three STs, MGT2 ST1-MGT3 ST89-MGT4 ST66, described 99% isolates of this genotype. In 1993, newer isolates with the *ctxB1-tcpA*^El Tor^ variant displaced isolates with the earliest *ctxB-tcpA* genotype and were sampled in almost all remaining years (typed as MGT3 ST13, ST19, ST133, ST137, and ST164). After 2010, the majority of isolates were *tcpA*^A226G^ with either *ctxB1* or *ctxB7*. MGT separated isolates of these genotypes. MGT3 ST6 described 97% of *ctxB7- tcpA*^A226G^ isolates, while MGT3 ST5 and MGT4 ST40 described 93% of *ctxB1-tcpA*^A226G^ isolates. In summary, the frequencies of the three genotypes were identified by *in silico* screening and were described by 33 nonoverlapping MGT STs.

## DISCUSSION

Cholera continues to threaten public health in developing countries across the globe (9, 18, 19). Outbreaks spread rapidly, and newer genomic technologies are required to process large volumes of data to identify and track transmission. In this study, we applied the novel genomic approach Multilevel Genome Typing (MGT) to V. cholerae, enabling the study of both long- and short-term epidemiology while providing a consistent and standardized nomenclature for isolates. The MGT scheme consists of eight MLST schemes named “levels,” which increase sequentially in number of loci and typing resolution (Fig. 1). The eight levels were filled with species core loci (MGT1 to MGT7) and 7P core loci (MGT8). The multiple levels offer flexibility in typing resolutions. The lowest MGT1 is the classic seven-gene MLST (9), and MGT7 and MGT8 represent the cgMLST schemes for the species and for the 7P, respectively. Locus selection methods for MGT2 to MGT4 and MGT5 to MGT6 differ. The lower MGT2 to MGT4 levels have loci that assign STs to reflect 7P phylogenetic structure (Fig. 2). Additionally, MGT2 to MGT4 have loci that only differ in non-7P isolates. MGT5 and MGT6 loci are randomly selected species core loci with expected mutation rates similar to that of the species core genome.

Using MGT, we analyzed all publicly available V. cholerae isolates (*n* = 5,771) and assigned each isolate a string of eight STs (one per MGT level). The loci filling the MGT levels are unchanging and define STs that can be directly compared between investigations. All assigned MGT identifiers are publicly available in the MGT database (https://mgtdb.unsw.edu.au/). The MGT web resource enables the assignment of MGT types to newly sequenced isolates. The MGT database is a centralized platform for standardized classification of V. cholerae species isolates.

### Long- and short-term seventh pandemic epidemiology by MGT.

Advances in DNA sequencing allow large numbers of high-quality genomes to be generated (20). Phylogenetic analysis of 7P isolates has examined both large- and small-scale genomic relationships (3, 6). However, even though phylogenetics can utilize the high level of detail offered by comparing thousands of core SNPs from whole-genome sequencing data, limitations exist. First, a lack of standardized methods prevents establishing transferable naming systems to describe lineages and/or clades of a phylogeny. Second, as new genomic data becomes available, phylogenetic relationships must be recalculated to include such data. As a result, naming systems have been developed within individual studies that are difficult to transfer between studies and apply to new data sets. For example, assigning any newly sequenced isolates to one of the three waves (lineages) is not straightforward without reanalyzing the relationships of the isolates from different waves (7, 9).

MGT allows standardized classification for the entire 7P. MGT2 to MGT4 STs reflect the 7P population structure (Fig. 2 and 3). Unlike phylogenetic analysis, these STs can be directly compared to newly sequenced isolates without recalculating genetic relationships for the complete data set. MGT2 to MGT4 STs capture both large-scale three-wave intercontinental transmission, as well as smaller groups causing outbreaks, such as the 2016 Yemen outbreak (3, 6). We propose a standardized nomenclature for the three waves of intercontinental transmission, namely, waves one, two, and three as MGT2 ST1, ST2, and ST3, respectively. This MGT nomenclature can further divide MGT2 STs (waves) to identify outbreak groups such as MGT4 ST12 for the Yemen outbreak (3, 6, 7, 15, 21).

MGT8 offered the highest-resolution typing for 7P isolates (Fig. 1) and has only marginally lower discriminatory power than SNP-based analysis (see Fig. S3 in the supplemental material). Additionally, as part of the MGT system, MGT8 has the advantage of a stable nomenclature, which is not defined in SNP-based analyses. MGT provides a heuristic alternative to phylogenetic analysis. The multilevel system provides close approximation to phylogenetic divisions and has the added advantage of rapidly typing large data sets. MGT is the first 7P typing tool that offers a range of typing resolutions and groups isolates with a standardized nomenclature that is transferable between public health or research laboratories worldwide.

### Application of MGT to non-7P isolates.

Since 1961, seventh pandemic (7P) isolates have been the major cause of cholera disease (22). However, during this period, toxigenic non-7P isolates have caused sporadic cases that often present milder symptoms (23). This includes non-7P O1 isolates and non-O1/non-O139 isolates (23, 24). MGT also classified non-7P isolates (Fig. 4). A V. cholerae cgMLST study recently reported a threshold of 133 allelic differences to define sublineages within the species (12). The selected threshold grouped isolates similarly to seven-gene MLST typing, allowing this single system to provide both low- and high-resolution classification. However, currently, no additional thresholds have been defined that could further divide isolates within the same sublineage. We show here that MGT offers better flexibility. The MGT1 to MGT7 levels sequentially divided the non-7P isolates (Fig. 4). Using the largest MGT1 type, ST75, as an example, MGT2 defined continent-associated STs (Fig. 4A) and MGT3 defined region-associated STs (Fig. 4B). Thus, V. cholerae MGT is applicable to non-7P clades with the same utility as that within the 7P.

### Stable and high resolution typing of lineages within the Haiti outbreak.

In 2010, cholera was reported in Haiti for the first time in over a century (16). Cholera continued to spread across Haiti and, by 2014, caused nearly 700,000 cases (25). SNP-based phylogenetic analyses resolved the Haiti outbreak origins by clustering Haitian and Nepalese isolates that differed by only two core genome SNPs (17). MGT reconfirmed the close relationships between the Nepalese and Haitian isolates, with MGT4 ST11 representing the Haiti outbreak clade that included isolates from both these countries, thus, demonstrating MGT as a valuable outbreak detection tool (Fig. 5). Prior to MGT analysis, a V. cholerae cgMLST scheme was used to provide a standardized name for the Haitian outbreak clone (12).

However, these isolates were grouped using 45 SNPs specific to the Haiti outbreak (26). While SNPs worked as a standardized system for clustering within the Haiti outbreak, these SNPs are not easily expandable for clustering the remaining 7P isolates. MGT4 ST11 provided the first standardized name for the Haitian outbreak that is part of a larger standardized nomenclature for the 7P and, more broadly, for the V. cholerae species.

During the Haiti outbreak, the most commonly used typing method, pulsed-field electrophoresis, was unable to distinguish isolates from within Haiti (8). The available methods were unable to easily and consistently incorporate new genomic data or identify emerging Haitian subpopulations as they arose. MGT5 was able to describe the evolution of the Haitian cholera outbreak (Fig. 5). Between 2010 and 2017, three distinct clades within Haiti were described by MGT5 ST30, ST1748, and ST1752 (Fig. 5B). MGT5 STs revealed that continual Haitian outbreaks were not caused by reintroduction, but instead by evolution of the outbreak clone. These three clades sequentially arose after initial outbreak reports in 2010, which was also confirmed by phylogenetic and temporal data (Fig. S4). Here, MGT5 identified valuable evolutionary information within a national outbreak.

### Seventh pandemic virulence determinant characterization using MGT.

During the 7P, newly evolved *ctxB and tcpA* variants have been characterized during epidemiological investigations (27, 28). In this study, *ctxB* and *tcpA* genotypes were identified from genomic sequences. MGT STs further described individual *ctxB* and *tcpA* and combined *ctxB*-*tcpA* genotypic frequencies across the 7P (Fig. 6). Isolates of the same genotype were best grouped by STs from multiple levels, and 33 STs from MGT2 to MGT4 on average described 97% of isolates per genotype. Additionally, single-level MGT STs described genotype trends, which has the advantage of simpler interpretation. However, we recommend using MGT STs from multiple levels, as they offer higher specificity.

Changing *ctxB*, *tcpA*, and *ctxB*-*tcpA* allelic frequencies have been used to track new isolates of the 7P (27). In Calcutta (India), surveillance of *tcpA* variants between 2004 and 2012 showed a shift in isolates that carried *tcpA*^A226G^ from *tcpA*^El Tor^ (29). These isolates also lacked the earliest 7P *ctxB3* and carried either a *ctxB1* or *ctxB7* variant. MGT STs were the first stable identifiers to describe the clades underlying changing frequencies of *ctxB*, *tcpA*, and *ctxB-tcpA* genotypes (Fig. 6). Focusing on *ctxB* variation, seven MGT STs from MGT3 and MGT4 grouped *ctxB1*-carrying isolates that characterized the introduction of the sixth pandemic *ctxB* into the 7P (Fig. 6A) (27). Classifying *ctxB1* isolates is of clinical significance. In Thailand, from 1999 to 2002, isolates with the *ctxB1* variant were associated with a resurgence in cholera-related hospitalizations with more severe symptoms (30). For *tcpA* variation, MGT2 ST3 described *tcpA*^A226G^-carrying isolates that in later years became the dominant *tcpA* variant (Fig. 6B). The *tcpA*^A226G^ variant is characteristic of 2010 Haiti outbreak isolates that have been linked through phenotypic analysis to “hypervirulent” isolates (31). Additionally, we showed MGT STs grouped isolates of the same *ctxB*-*tcpA* genotypes (Fig. 6C). MGT3 ST6 typed 97% of ctxB7- *tcpA*^A226G^ variants, the same isolates that caused the worst outbreak in modern history, the 2016 Yemen outbreak (6). The benefit of describing *ctxB*, *tcpA*, and *ctxB*-*tcpA* genotypes by MGT is that groups of isolates are classified by a transferable nomenclature that facilitates communication between investigations. The MGT database offers a centralized resource for global *in silico* genotyping of *ctxB* and *tcpA* combinations that can be expanded to include additional *ctxB* and *tcpA* variants and/or alternative virulence factors. Submitting newly sequenced isolates to the MGT database will continue to quantify changes in genotypic frequencies, and their trends can be simply communicated using MGT STs.

### Conclusion.

In this study, we developed an eight-level MGT scheme for characterization of the V. cholerae population. These levels offer flexibility in typing resolutions that continually divides isolates into smaller subgroups. Initially, we analyzed a 7P data set (*n* = 4,770 isolates). MGT2 recapitulated the three waves of the 7Ps, and MGT3 identified continent-specific and multicontinental lineages. To examine the utility of MGT at higher resolutions, the Haiti outbreak was examined. MGT5 identified three dominant STs that sequentially evolved and spread after initial outbreak reports in 2010. MGT also classified non-7P isolates. MGT successfully typed 97% of non-7P isolates and was able to define epidemiologically informative subtypes within a large clade. Finally, MGT was used to describe groups of isolates with the same *ctxB*, *tcpA*, and *ctxB-tcpA* genotypes. Thirty-three STs from MGT2 to MGT4 quantified changing frequencies of *ctxB*, *tcpA*, and *ctxB-tcpA* genotypes across the 7P. MGT defines a unique scalable standardizable typing scheme that can produce a stable nomenclature for surveillance of V. cholerae. All assigned MGT identifiers for this study are publicly available in the MGT database. As genome sequencing becomes more common, the need for a standardized, rapid, and simple method for identifying and communicating isolate relationships will only become more pressing. MGT provides such a system. The MGT database can quickly provide valuable information to better track the initial and ongoing spread of future 7P and non-7P outbreaks. We present MGT as a new standardized system for both long- and short-term V. cholerae epidemiology.

## MATERIALS AND METHODS

Methods are summarized in a flow diagram (see Fig. S1 in the supplemental material).

10.1128/mSystems.00134-21.4 FIG S1 Flow diagram overview of methods. The four stages for Multilevel Genome Typing (MGT) design and application. Download FIG S1, EPS file, 1.3 MB. Copyright © 2021 Cheney et al. 2021 Cheney et al. https://creativecommons.org/licenses/by/4.0/ This content is distributed under the terms of the Creative Commons Attribution 4.0 International license.

### Data set curation.

Sequence Read Archive Illumina whole-genome sequencing data for 8,039 V. cholerae genomes were downloaded using NCBI-genome-download (32). Raw reads were trimmed with Trimmomatic v0.36 and assembled using SPAdes v3.7 (33, 34). High-quality assemblies were then selected using statistics generated by QUAST v5.0.2 (35). The following quality thresholds were applied to assemblies: between 3.9 and 4.1 million bp long, GC content between 40% and 55%, fewer than 500 contigs, an *N*_50_ greater than 3,000, and more than 2,500 predicted genes. Assemblies were removed if Kraken v0.10.5-beta identified less than 75% V. cholerae DNA (36). Genomes were annotated using Prokka v1.7 (37). From the remaining assemblies (*n* = 5,754), a 7P (ST69 and ST515)-exclusive data set (*n* = 4,770 genomes) was identified by MLST v2.11 (38).

### Core genome definition.

To fill the MGT levels, Roary v3.5 was used to define core genes for the V. cholerae species and exclusively for the V. cholerae 7P (39). Additionally, 7P core intergenic regions (IGRs) were defined using Piggy v1 (40). Paralogues were removed using an in-house Python script (Text S1, “Supplementary Methods 1.1”).

### MGT design.

MGT2 to MGT6 loci were selected from the species core genome and contained mutually exclusive locus sets. The selection of loci between MGT2 to MGT4 and MGT5 to MGT6 differed. MGT2 to MGT4 loci were selected to separate lineages based on 7P phylogenetic structure. Initially, MGT8 (cgMSLT) alleles were identified for the 7P data set, and then a complete 7P phylogeny was created using MGT8 allelic profiles. The complete 7P phylogeny was used to identify major 7P lineages, and 80 isolates from basal branches of these lineages were selected for a representative 7P phylogeny. An in-house Python script identified alleles of core loci that were unique to desired lineages of the representative 7P phylogeny (Text S1, “Supplementary Methods 1.2”). MGT5 to MGT6 loci were randomly selected from a pool of species core loci based on typeability and neutrality (Text S1, “Supplementary Methods 1.3 to 1.4”).

### MGT processing.

All assemblies (*n* = 5,771 genomes) were typed by the newly developed MGT levels. Allelic differences were calculated for each level, and all isolates were assigned an ST at each level. Allele and ST calling were performed using established MGT pipelines (13).

### *In silico* genotyping.

A BLAST-based algorithm was developed for reporting marker genes across the 7P pandemic (*in-silico*-genotyper, https://github.com/liamcheney/MGT-Seventh-Pandemic) (27). All genomes were queried against a database of virulence genes (*ctxB* and *tcpA*). Alleles for these genes were collected from UniProt (27). Genes were reported as present if an allele in the database matched exactly (100% length and identity). MGT STs from each level were used to describe groups of isolates with the same combination of *ctxB-tcpA* alleles (referred to as genotypes). Assigning genotypes to STs required a minimum size of 10 isolates in the given ST and at least 75% of the isolates in the given ST to have the same genotype. Isolates with particular genotype were assigned exclusively to one ST at a given MGT level to prevent overlapping STs from different levels describing the same isolates. These relationships were visualized in Tableau v9.1 (41).
