# Supplementary material for: Multilevel Genome Typing Describes Short- and Long-Term Vibrio cholerae Molecular Epidemiology
Source: mSystems. 2021 Aug 24;6(4):e00134-21. doi: 10.1128/mSystems.00134-21 (PMC8407458; doi:10.1128/mSystems.00134-21)
Supplement: TEXT S1 [file msystems.00134-21-s0001.docx]

Supplementary Materials

# Supplementary Methods

## Core Genome Definition

### Creating the Species and Seventh Pandemic Datasets

The processed genomes were separated into two datasets. Genomes for both of these datasets were chosen based on their genetic diversity and were sequenced from a range of environmental and large population studies. First was the species dataset which contained 1936 *V. cholerae* genomes. Second was 1610 *V. cholerae* seventh pandemic genomes. A seven gene MLST scheme was used for identifying the entire *V. cholerae* seventh pandemic as ST69.^1^ Using BLASTN ^2^, only genomes with 100% nucleotide similarity with all seven loci from the MLST scheme were added to the seventh pandemic dataset.

### *V. cholerae* and Seventh Pandemic Core Gene Definition

Core genes for the two datasets were defined using Roary (v3.5.7).^3^ The two adjusted arguments were: the minimum percentage blast nucleotide identity (BNI) for two genes to be assigned as core, and the minimum percentage of all genomes a gene was present in for the gene to be called as core. To determine the appropriate level of BNI for both datasets, a series of analysis starting with 100% BNI and reducing in 1% increments to 90% BNI was performed. Additionally, core genes with duplicates (paralogues) in any genome were not selected as part of either datasets core genome. Duplicate core genes were removed using an inhouse python script (Script 1, <https://github.com/liamcheney/MGT-Seventh-Pandemic>). Core gene locus tags were assigned according the *V. cholerae* N16969 reference genome.

### Core Intergenic Region Definition

Using Piggy (v3.0)^4^, the core IGRs for only the seventh pandemic dataset were defined. Similar to Roary, a series of core IGR analyses were curved out and varied in BNI ranged from 100 – 90% and decreased in 1% increments. For all analysis, intergenic regions (IGRs) ranged in length between 100-1000 bps. All remaining arguments were default.

## Seventh Pandemic Population Structure

Phylogenetic reconstruction was used to resolve the seventh pandemic population structure. Genetic distances for the seventh pandemic dataset (*n= 4770 genomes*) were calculated using allelic differences from the seventh pandemic core genome. This required curating a preliminary a core genome MLST database consisting with a single seventh pandemic core genome level. Genetic differences based on seventh pandemic core genome alleles were used to create neighbour joining phylogenies visualised using a Grapetree v1.0 and generated by rapidNJ.^5^ The seventh pandemic dataset phylogeny was further summarised by a maximum likelihood representative phylogeny of 80 isolates located from basal branches. Snippy v4.0 extracted core SNPs and RecDetect v6.1 removed core SNPs affected by recombination.^6,7^ RaxML v8.1 calculated genetic distances with defaults settings and 1000 bootstrap replicates.^8^

Additionally, as a part of a MGT case study the population structure of the Haiti outbreak was phylogenetically reconstructed. This involved a subset of 224 Haiti outbreak isolates that had both temporal and geographic metadata. Reconstruction followed methods used to create the representative seventh pandemic phylogeny. The phylogeny was annotated with metadata for year and country of isolation, and MGT4 and 5 STs generated in later methods. MGT STs and geographic metadata were visualised using Tableau v9.1.^9^

## Calculating the number nucleotides for MGT5-MGT6

After assigning the species core gene preferences, the number of nucleotides for each MGT2 – MGT6 scheme was found. The MGT was designed so schemes MGT2, 3, 4, 5 and 6 were scaled to 100, 20, 10, 5 and 1 years, respectively. To find the amount of DNA per scheme, the years for a single SNP to occur in the *V. cholerae* N16969 reference was calculated using the whole genome mutation rate.^10^ Then the amount of DNA producing one SNP over the schemes time period was used as the scheme’s size (bp). The below formula calculated the size of each MGT scheme in bp (*S*), using a mutation rate (*μ*) of 3.91 SNPs/year/genome, the *V. cholerae* reference genome length of 4,161,908 bp (*l*), and the time in years (*T*) which varied depending on the scheme (outlined above).

$$S=\frac{l}{m x T}$$

## Filtering Species Core Genes into MGT5-6

### Organisation of the Core Genome

Prior to typing the seventh pandemic using the MGT, the loci from both core genomes were organised into the eight MGT schemes. Schemes 1 to 4 and 7 to 8 did not require loci to be organised in a specific order. However, organising species core genes into schemes 5 to 6 relied on multiple stages outlined below.

### MGT5 – MGT6 Genes: Filtering Typable Genes

Filtering genes for MGT5 – MGT6 included testing typability (Script 2, <https://github.com/liamcheney/MGT-Seventh-Pandemic>). This script was part of the MGT pipeline and was used to determine the quality of allele assembly for each species gene across the 1936 species genomes used for core genome definition. Genes were either recorded as non-typable or typable. Non-typable genes were missing up to 20% sequence in >5% of the test genomes. typable genes were missing no sequence in >95% of the test genomes. Genes missing >20% sequence in >5% were removed.

Additionally, species core genes with tandem repeats and homopolymer tracts were identified using Tandem Repeat Finder ^11^ and an in-house python script (Script 3, <https://github.com/liamcheney/MGT-Seventh-Pandemic>), respectively.

### MGT5 – MGT6 Genes: Calculating d_N_/d_S_ Mutation Rate

Genes not under strong selection pressure were chosen the MGT5 – MGT6 schemes. The mutation rate (dN/dS) was calculated to indicate the balance of purifying (negative) to positive selection upon the species core gene. A subset of 50 genomes from the 1936 species genomes were chosen to reduce computation time. These genomes were typed as 32 different *V. cholerae* STs using MLST v2.11.^12^ For each species core gene, an inhouse python script (Script 4, <https://github.com/liamcheney/MGT-Seventh-Pandemic>) located the sequence across the subset and concatenated the sequences. Sequences with truncations or missing data (N’s) were excluded. Multiple sequence alignments were created using Muscle v3.8.31.^13^ Codon alignments were created with Pal2nal v14^14^ and per gene mutation rates were calculated using SNAP v2.2.1.^15^ When generating codon alignments used the bacterial translation table. The distribution of d_N_, d_S_ and d_N_/d_S_ rates were visualised using GraphPad Prism 7.0.

### MGT5 – MGT6 Genes: Qualitative Likelihood of Selection Pressure

In addition to calculating the d_N_/d_S_ rate of evolutionary pressure, a qualitative approach to identify the genes most likely to be under positive selection pressure was developed. This approach combined data from a range of Databases and Tools. Additionally, genes lengths and characteristics that commonly caused assembly problems were identified. These database and tools are shown in Supplementary Methods Table 1.

Supplementary Methods Table 1. Databases and tools used for identifying genes most likely to not be under positive selection pressure.

| **Database or Tool** | **Gene Characteristic** | **Reference** |
| --- | --- | --- |
| BioCyc Database | Excluded Locations: predicted in “Extracellular Space”, “Inner Membrane”, “Outer membrane”, “Periplasmic Space”, “Cell Wall or Pilus” | ^16^ |
| Pathosystems Resource Intergration Centre (PATRIC) | Excluded Functions: predicted “Virulence functional category”, “Cell wall and capsule, Phages”, Prophages”, “Transposable elements, Plasmid”, “Virulence, Disease and Defence” and/or “Motility and Chemotaxis” | ^17^ |
| Phaster | Phage Genes | ^18^ |
| Pred-TAT | Twin Arginine Transport (TAT) Regions | ^19^ |
| SignalP | Secretory Pathway | ^20^ |
| Roary | In Genus Core Genome | ^3^ |
| TMHMM | Transmembrane Helices | ^21^ |
| T3SS | Type III Secretion System (T3SS) | ^22^ |

### MGT5 – MGT6 Genes: Assigning Each Gene a Preference Number

Based on the multiple stages in sections 1.4.1 - 1.4.4, the species core genes were ranked into preferences 1-10. The criteria defining each preference are explained in The criteria for each preference are available . Yellow boxes indicate which criteria was changed to define that preference. Core genes recorded as “True” had the applied criteria. For example, a core gene “True” for “Excluded locations”, encoded for a protein which was localised in the excluded areas defined in section 1.4.3. Each gene was assigned once to the lowest preference to which it matched all criteria. The criteria for each preference are available in Supplementary Results Table 2.

Supplementary Results Table 2. Overview of Preference Design System

| **Preference** | **Seventh Negatives** | **Seventh Zeros** | **Species Negatives** | **Species Zeros** | **In Genus Core** | **In dS Fifty Percen** | **In dNdS Fifty Percen** | **In Phasta** | **In T3SS** | **Has TMH** | **Has Signal Peptides** | **Has pred-TAT** | **In Bio Cycle Excluded** | **In Patric Excluded** | **In Gene Size Ninety Percen** | **In dNdS Ninety Percen** | **In dS Ninety Percen** | **In Species Core** |
| --- | --- | --- | --- | --- | --- | --- | --- | --- | --- | --- | --- | --- | --- | --- | --- | --- | --- | --- |
| **1** | 0 | 0 | 0 | 0 | TRUE | TRUE | TRUE | FALSE | FALSE | FALSE | FALSE | FALSE | FALSE | FALSE | TRUE | TRUE | TRUE | TRUE |
| **2** | 15 | 5 | 15 | 5 | TRUE | TRUE | TRUE | FALSE | FALSE | FALSE | FALSE | FALSE | FALSE | FALSE | TRUE | TRUE | TRUE | TRUE |
| **3** | 15 | 5 | 15 | 5 | FALSE | TRUE | TRUE | FALSE | FALSE | FALSE | FALSE | FALSE | FALSE | FALSE | TRUE | TRUE | TRUE | TRUE |
| **4** | 15 | 5 | 15 | 5 | FALSE | FALSE | FALSE | FALSE | FALSE | FALSE | FALSE | FALSE | FALSE | FALSE | TRUE | TRUE | TRUE | TRUE |
| **5** | 15 | 5 | 15 | 5 | FALSE | FALSE | FALSE | TRUE | TRUE | FALSE | FALSE | FALSE | FALSE | FALSE | TRUE | TRUE | TRUE | TRUE |
| **6** | 15 | 5 | 15 | 5 | FALSE | FALSE | FALSE | TRUE | TRUE | TRUE | TRUE | TRUE | FALSE | FALSE | TRUE | TRUE | TRUE | TRUE |
| **7** | 25 | 10 | 25 | 10 | FALSE | FALSE | FALSE | TRUE | TRUE | TRUE | TRUE | TRUE | TRUE | TRUE | TRUE | TRUE | TRUE | TRUE |
| **8** | 25 | 10 | 25 | 10 | FALSE | FALSE | FALSE | TRUE | TRUE | TRUE | TRUE | TRUE | TRUE | TRUE | FALSE | FALSE | TRUE | TRUE |
| **9** | 25 | 10 | 25 | 10 | FALSE | FALSE | FALSE | TRUE | TRUE | TRUE | TRUE | TRUE | TRUE | TRUE | FALSE | FALSE | FALSE | TRUE |
| **10** | 25 | 10 | 25 | 10 | FALSE | FALSE | FALSE | TRUE | TRUE | TRUE | TRUE | TRUE | TRUE | TRUE | FALSE | FALSE | FALSE | FALSE |
| **11** | >25 | >10 | >25 | >10 | FALSE | FALSE | FALSE | TRUE | TRUE | TRUE | TRUE | TRUE | TRUE | TRUE | FALSE | FALSE | FALSE | FALSE |

Based on the results from 2.4.1– 2.4.5, the seventh pandemic core genes were ranked into preferences 1-11. The criteria defining each preference are explained in Supplementary Results Table 2. Yellow boxes indicate which criteria was changed to define that preference. Core genes recorded as “True” had the applied criteria. For example, a core gene “True” for “Excluded locations”, encoded for a protein which was localised in the excluded areas defined in section 2.4.5. Each gene was assigned once to the lowest preference it matched the criteria for.

### Organisation of Species Core Genes into MGT5 – MGT6.

The final method for organising the species core genes into MGT2 – MGT7 used an in-house python script (Script 5, <https://github.com/liamcheney/MGT-Seventh-Pandemic>). This script used schemes size, all previously assigned preferences and additionally required a minimum distance of nucleotide separation for genes within each scheme. For each scheme, genes were selected from a pool of species core genes meeting if located greater than a selected distance (b.p). If the pools summed size was greater than the schemes size, genes were randomly selected from the pool to fill the scheme. The excess genes were then available for selection in the next larger scheme. Once a gene was allocated, it was no longer available for re-selection. A trial and error method optimised the lowest gene preferences and greatest distances of separation for each scheme.

## In-silico Predicting *V. cholerae* Serogroups

An in-house python script was developed to predict *V. cholerae* seventh pandemic serogroups. Isolates were screened for variants of the *wb** region responsible for O-antigen synthesis.^23,24^ *rfbV* gene presence was associated with the O1 serogroup, and *wbfZ* for the O139 serogroup. Complete (100%) alignment length and nucleotide similarity predicted serogroup. The *V. cholerae* O1 N16916 *rfbV*, and *V. cholerae* O139 MO10 *wbfZ* were used.^10,25^

# Supplementary Results

## Defining the Loci filling the MGT schemes

### *V. cholerae* Species Core Genes Filled MGT2-8

MGT classification compares shared loci (core loci) between a set of analysed genomes. Thus, the schemes of the MGT must be filled using core loci for accurate typing of the *V.* *cholerae* seventh pandemic. The *V. cholerae* species and seventh pandemic core genomes were defined to use their loci in the MGT schemes. The first stage of this project involved identifying the *V. cholerae* species core genes. These core genes were later used to fill MG2-MGT8.

The species core genome was defined using the quality filtered and genetically diverse 1936 *V. cholerae* genomes. Defining the core genes involved choosing the highest blast nucleotide identity percentage (BNI) shared between core genes, which did not significantly reduce the number of core genes. Selecting a BNI of 100% would indicate that all core genes would have no nucleotide diversity across the species. This would substantially reduce the number of core genes. Lowering the BNI below 90% could erroneously add genes to the core genome not located across the dataset.^3^

To determine the highest BNI not dramatically lowering the number of core genes, the species core genes were calculated using a series of BNI. Beginning at 100% BNI and reducing by 1% increments to 90% BNI, the number of core genes was calculated for each BNI. The number of species core genes increased from 769 to 2530 when reducing BNI from 100% to 90%, respectively (Supplementary Figure 2A). A plateau of core gene increase was observed at 96% BNI with 2495 core genes. Reducing the BNI below 96% did not cause a significant increase in the number of core genes. For this reason, the 2495 species core genes defined at 96% were selected for the species core genome (Supplementary Figure 2A, outlined in the grey box). However, after removing paralogous genes the number of core genes reduced to 2495. The defined 2495 species core genes were expected be present across the entire *V. cholerae* species and were later used to fill MGT2-MGT8.

### The *V. cholerae* Seventh Pandemic Core Genes filled MGT8

Definition of *V. cholerae* seventh pandemic core genes were similar to the process explained in Supplementary Results 2.1.1. Accurate MGT classification relied on filling the schemes with loci located across the entire seventh pandemic. Hence, the core genes of the seventh pandemic dataset were defined using Roary. The seventh pandemic genome set was a subset of 1610 seventh pandemic genomes. The seventh pandemic only genomes were confirmed using a seven-gene MLST scheme.^1^

Explained in Supplementary Results 2.1.1, the process for selecting BNI in species core gene definition was re-used. A series of BNIs were used when calculating the seventh pandemic core genes. In increments reducing by 1%, the number of seventh pandemic core genes was determined between 100% BNI and 90% BNI (Supplementary Figure 2B, shown in red). Reducing the BNI from 100% to 90% resulted in core genes increasing from 2321 to 3042 respectively. The number of core genes begun to plateau at 3032 with 96% BNI. Further reducing the BNI below 96% did not significantly increase core gene number. A BNI of 96% defining 3032 core genes included paralogs (duplicates). After removing paralogs 2854 non-duplicate core genes represented the seventh pandemic core genes (Supplementary Figure 2B, outlined in the grey box). Maintaining a high BNI was essential to define core genes found across the entire seventh pandemic. The 2854 core genes were identified using a high BNI, indicating they were suitable for classification with the MGT. The seventh pandemic core genes were added to MGT8.

### MGT8 Included *V. cholerae* Seventh Pandemic Core Intergenic Regions

The loci filling the schemes of the MGT were not restricted to genes. The core IGRs shared between seventh pandemic strains, increased the number of loci for comparing strains and provided a higher MGT typing resolution. The core IGRs were only defined for the seventh pandemic dataset.

A similar approach to species and seventh pandemic core gene definition was applied when determining the core IGRs of the seventh pandemic. This involved defining the core IGRs using a range of BNIs, to achieve the highest BNI which did not significantly reduce the number of core IGRs. Lowering the BNI from 100% to 90% caused a significant increase in the number of core IGRs, from 206 to 915 respectively (Supplementary Figure 2C, shown in red). A BNI of 91% was selected to define the 905 core IGRs (Supplementary Figure 2C, shown in the grey shaded box). A lower BNI was not selected, as the number of core IGRs did not significantly increase. Indicating the majority of core IGR diversity was captured at 91% BNI. The 905 core IGRs were added to the seventh pandemic core genome and used in MGT8, which was the highest resolution scheme for MGT classification.

## Comparing Allele and SNP Pairwise Distances

The allele profiles for 300 representative seventh pandemic isolates were compared to the calculated SNP pairwise distances. Between all isolates and the N16916 reference 48,346 SNPs were identified and 23% (910,982/48,346) were further removed as they were located in Phage regions, mobile genetic elements or recombinant regions. The frequency of pairwise distances for allele and SNP based methods were shown to both be normally distributed (Supplementary Figure 3A). SNP distances were normalised by 77% to reflect the percentage the core genome represented of the N16916 reference. After normalising, the mean pairwise distance for each method was almost identical. Allele and SNP pairwise distances were on average 71 alleles and 70 SNPs, respectively. The concordance between allele and SNP calling was visualised (Supplementary Figure 3B). Comparing pairwise distances to a theoretical one-to-one ratio (Supplementary Figure 3B, hashed grey line) showed SNP distances were greater than allelic. More than 95% of isolates varied by less than 200 SNPs and 160 alleles. A ratio of SNPs to alleles showed that for every defined SNP there were 0.81 alleles defined. Indicating that SNP resolution was higher than allele-based typing.

## The Calculated MGT2-6 Scheme Sizes

Before assigning the species core genes to MGT2 – MGT6 the target sizes of the schemes was calculated. The sizes of the MGT2 – MGT6 schemes were calculated in relation to desired time periods (shown in Supplementary Results Table 3). Even though the schemes represented different time periods, each scheme aimed to separate isolates by a single SNP. Therefore, the size of each scheme was the amount of DNA required for a single SNP to occur for that schemes designated time. The mutation rate used for calculating the schemes sizes were based on the *V. cholerae* N16916 whole genome mutation rate.^10^

An inverse trend between the schemes desired years and sizes was seen, where MGT2 was 10,329 bp in size and represented 100 years, while MGT6 was 1,032,875 bp in size and represented one year (Supplementary Results Table 3). This was expected as the size of each scheme reflected the amount of DNA required for a single SNP to occur in the desired timeframe. Therefore, assuming the genes in a scheme were not under positive selection, the smaller amount of DNA in lower schemes would take a longer time for a SNP to appear. While by random chance a single SNP would occur in shorter time periods in a larger amount of DNA. These results were used for setting scheme size limits when organising the species core genes into MGT2 to MGT6.

Supplementary Results Table 3. The calculated size for MGT schemes 2 to 7.

| **MGT Scheme** | **Scheme Time Period (years)** | **Size of Scheme (b.ps)** |
| --- | --- | --- |
| MGT2 | 100 | 10329 |
| MGT3 | 20 | 51644 |
| MGT4 | 10 | 103287 |
| MGT5 | 5 | 206575 |
| MGT6 | 1 | 1032875 |

## Organisation of Species Genes into MGT5-6

Species core genes were selected for MGT5-6. Instead of randomly selecting species core genes, a preference system allocated genes into MGT5-6. This was important as individual genes in the smaller MGT5 had a greater impact when assigning STs. The preference system provided consistency when assigning STs between lower schemes by not selecting genes more likely to create allelic variance.

The preference system identified the species core genes reliable for classification (referred to as typability) and not under positive selection pressure. Identifying genes not under positive selection pressure combined results from two approaches: a quantitative method of calculating d_N_/d_S_ ratios (balance of negative and positive selection pressure), and a qualitative method which combined results from a range of databases and tools. These databases and tools identified species core genes encoding proteins interacting with the host which are more likely to be under positive selection to change.

### MGT5 to MGT6: Identifying Typable Species Core Genes

Preferences were assigned to each species core gene based on typability which reflected how reliably a gene was located across the species. A typable gene was defined as having less than 20% missing sequence in greater than 95% of a test dataset of 1936 species genomes. Non-typable genes were missing more than 20% sequence in more than 5% of the test genomes. A significantly lower number of genes were non-typable compared to typable, with 22 and 2277 respectively. Of the 2277 typable genes, 1836 were identified as missing no sequencing for the entire test dataset. The small amount of non-typable genes indicated quality filtering steps were effective in removing poor quality assemblies and the species core genes were accurately identified.

An additional method of inferring typability involved identifying core genes with repeat regions. The species core genes were tested for the presence of homopolymer tracts and tandem repeats. A total of 27 and 15 core genes were shown to have homopolymer and tandem repeats, respectively.

### MGT5 to MGT6: Roary Defined and PubMLST Species Core Genes

During publishing the seventh pandemic MGT a *V. cholerae* core genome was released through PubMLST. The overlap of species core genes defined by Roary in this study, and the PubMLST core genome were selected when filling MGT5 – MGT6. This overlap included 2344 species core genes.

### MGT5 to MGT6: Species Core Genes Selection Pressures

For each of the 2998 species core genes a codon alignment was created to calculate the d_N_/d_S_ ratio. Each codon alignment attempted to extract sequences from a diverse dataset of 55 *V. cholerae* genomes. Quality filtering of the alignments removed sequences with truncations and missing data. d_N_/d_S_ ratios for core genes were calculated if the respective alignment contained 54/55 (99%) sequences. For 2137 of the 2998 core genes a d_N_/d_S_ ratio was calculated. The d_N_/d_S_ ratios were distributed between 0 and 0.75. The distribution was strongly left skewed towards d_N_/d_S_ = 0, indicating the species core genes were predominately under negative selection pressure. The mean d_N_/d_S_ was 0.69 (2 dp) with a minimum value of 0 and a maximum of 0.75. Later the genes were MGT5-MGT6 were selected. Species core genes with a d_N_/d_S_ in the 50^th^ percentile were selected for MGT5 and the remaining were used for MGT6. Additionally, the same approach was used to separate species core genes using d_N_. Genes with a lower d_N_ were selected for MGT5 and genes with a higher d_N_ were in MGT6. Further explained below.

### MGT5 to MGT6: Distribution of Species Gene Lengths

The length of genes was considered when choosing species core genes for MGT5 – MGT6. Genes with longer lengths are more likely to accumulate mutation by random chance purely based on their size. The average gene length was 897 bps with the most common gene sizes between 600-800 bps. While selecting the species core genes for MGT5 – MGT6, all genes were within the 90^th^ percentile.

### MGT5 to MGT6: Identifying Genes Most Likely Not Under Positive Selection Pressure

The next criteria for preferences accounted for the likelihood of a species core gene to not be under positive selection pressure. A qualitative approach to identify genes not under positive selection combined results from a range of databases and tools (Supplementary Results Table 4). True and false results for the 2495 species core genes were recorded. A true result indicated the analysed gene returned a positive result for the database or tools main function. For example, a gene true for the THMM tool, encoded for a transmembrane helix and a gene true for Phaster was annotated as a Phage Gene (Supplementary Results Table 4). The results from these databases and tools were used in the preferences for identifying the core genes most likely not under positive selection pressure. Species genes also in the genus core genomes, not in excluded locations and functions categories, not involved with: phage development, the secretory pathway, type III secretion, TAT, were most likely not under positive selection pressure.

Supplementary Results Table 4. Qualitatively results for species core genes using databases/tools.

| **Database or Tool** | **False** | **True** |
| --- | --- | --- |
| Roary: *Vibrio Genus Core Genome* | 1232 | 1766 |
|  |  |  |
| Patric: Excluded Functions * | 1750 | 548 |
| Biocycle: Excluded Locations ** | 2190 | 108 |
|  |  |  |
| Phaster: Phage Gene | 2286 | 12 |
| SignalP: Secretory Pathway | 1941 | 357 |
| T3S: Type III Section System | 1950 | 348 |
| THMM: Transmembrane Helix | 1712 | 586 |
| Pred-TAT: Twin-Arginine-Transport | 1508 | 790 |

* Excluded Functions were: “Virulence functional category”, “Cell wall and capsule, Phages”, Prophages”, “Transposable elements, Plasmid”, “Virulence, Disease and Defence” and “Motility and Chemotaxis”.

** Excluded locations were: “Extracellular Space”, “Inner Membrane”, “Outer membrane”, “Periplasmic Space”, “Cell Wall or Pilus”.

### MGT5 to MGT6: Assigning preferences to the species core genes

The preference system divided the species core genes into groups reflecting typability, selection pressure, length and likelihood of not being under positive selection pressure. The typability indicated the genes with the less prone to sequencing and assembly errors. The calculated d_N_/d_S_ rates indicated the selection pressure of a species core gene. The gene lengths were used for selecting genes of an average length and the likelihood of selection pressure was qualitatively measured using a combination of tools and databases. The chosen databases and tools identified genes encoding proteins interacting with the host. Proteins interacting with the host are more likely under positive selection pressure as they may induce adaptations.^26,27^ The species core genes were all assigned a preference based on results from Supplementary Results 2.4.1-2.4.5. Preference 12 was the most common and was assigned to 451 core genes. The lowest preferences had the most stringent criteria. The 70 species core genes in preference 1 were most likely not under positive selection, and met the criteria: not missing any data across the 1936 species core genomes, in the genus core genome, in 50% percentile of d_S_ distribution, in 50% percentile of d_N_/d_S_, not a phage gene, not involved in type III secretion, has no transmembrane regions, not involved in secretory pathways, no tandem repeats, no homopolymer tracts, not in excluded locations or function categories and within 90% percentile of gene lengths.

The first preferences 1,2 and 3 had 70, 21, 54 core genes, respectively. These preferences had lower amounts of genes compared to preferences 4,5,6 and 9-13. The lower 1,2 and 3 preferences had more strict criteria for selecting genes not under positive selection pressure and explains the lesser number of genes in these preferences. All species core genes not assigned to preferences 1-12 were allocated to preference 13 by default. The 200 core genes in preference 12 were not used to fill the MGT5 – MGT6.

### Organisation of Species Core Genes into MGT5-6

Finally, species core genes were organising into MGT5-6. A total of 507 genes from preferences 1-8 with a 1kb minimum distance of separation were allocated to MGT5 (Supplementary Results Table 5). After meeting the MGT5 scheme size of 516,437 b.ps, MGT6 was allocated genes. 954 core genes were allocated to MGT6 which met the size requirement of 1,032,875 b.ps. Selecting enough genes to fill MGT6 required no minimum distance of separation, and genes from preferences 1-10. For MGT5-6 genes were randomly selected from the designated preferences. Genes from the lowest preference were selected before selecting genes in higher preferences.

Supplementary Results Table 5. Criteria for allocating species core genes in schemes MGT5-6.

| **MGT Scheme** | **Size (bp)** | **Preferences to Select Genes From** | **Minimum Nucleotides Between**  **Each Gene (kilobases)** | **Core Genes per Scheme** |
| --- | --- | --- | --- | --- |
| MGT5 | 516437 | 1-8 | 1000 | 507 |
| MGT6 | 1032875 | 1-10 | 0 | 954 |

## O1/O139 In-Silico Prediction

The serogroup of 4,770 seventh pandemic isolates was predicted in-silico. The presence of either *rfbV* or *wbfZ* genes were detected in 99.9% (4769/4770) of isolates. The large majority were predicted as O1 serogroup (4219/4769), and 12% (550/4769) were predicted as O139 serogroup. A single isolate (accession: ERR2632175) was unable to have either *rfbV* or *wbfZ* genes located.

## Single MGT Level Description of Virulence Genotypes

The single MGT level that best grouped 2015 isolates with the same *ctxB*, *tcpA* and *ctxB*-*tcpA* genotypes was investigated. STs assigned by each MGT level were characterised genotype specific, not-specific and minor. Overall, genotype specific STs from MGT3 grouped on average 94% of isolates for each genotype (Supplementary Figure 6A). MGT1-2 STs were unable to specifically group isolates based on genotype and the majority of MGT1-2 STs were characterised as not genotype specific (Supplementary Figure 6B-D, shown in light green). Likewise, the majority of MGT5-8 STs were unable to describe genotypes and MGT5-8 STs were mainly minor (assigned to less than 10 isolates) (Supplementary Figure 6B-D, outlined in green). MGT3-4 were the only levels with majority genotype specific STs (Supplementary Figure 6B-D, shown in dark green). In total, MGT3 assigned 36 STs that grouped isolates based on genotype (12 per genotype) (Supplementary Figure 6B-D, marked by asterisk). For each genotype these STs on average described 94% of isolates.

# References

1 Luo Y, Ye J, Jin D, et al., (2013), Molecular analysis of non-O1/non-O139 *Vibrio cholerae* isolated from hospitalised patients in China, BMC Microbiology, <https://doi.org/10.1186/1471-2180-13-52>.

2 Altschul SF, Gish W, Miller W, Myers EW, Lipman DJ, (1990), Basic local alignment search tool, Journal of molecular biology, <https://doi.org/10.1016/s0022-2836(05)80360-2>.

3 Page AJ, Cummins CA, Hunt M, et al., (2015), Roary: rapid large-scale prokaryote pan genome analysis, Bioinformatics, <https://doi.org/10.1093/bioinformatics/btv421>.

4 Thorpe HA, Bayliss SC, Sheppard SK, Feil EJ, (2018), Piggy: a rapid, large-scale pan-genome analysis tool for intergenic regions in bacteria, Gigascience, <https://doi.org/10.1093/gigascience/giy015>.

5 Zhou Z, Alikhan NF, Sergeant MJ, et al., (2018), GrapeTree: visualization of core genomic relationships among 100,000 bacterial pathogens, Genome Research, <https://doi.org/10.1101/gr.232397.117>.

6 Hu D, Liu B, Wang L, Reeves PR, (2019), Living Trees: high-quality reproducible and reusable construction of bacterial phylogenetic trees, Molecular Biology and Evolution, <https://doi.org/10.1093/molbev/msz241>.

7 Seemann T. Snippy: fast bacterial variant calling from NGS reads 2015 [Available from:<https://github.com/tseemann/snippy>.

8 Stamatakis A, (2006), RAxML-VI-HPC: maximum likelihood-based phylogenetic analyses with thousands of taxa and mixed models, Bioinformatics, <https://doi.org/10.1093/bioinformatics/btl446>.

9 Deadorf A, (2016), Tableau (version. 9.1), Journal of the Medical Library Association : JMLA, <https://doi.org/10.3163/1536-5050.104.2.022>.

10 Duchêne S, Holt KE, Weill F-X, et al., (2016), Genome-scale rates of evolutionary change in bacteria, Microb Genom, <https://doi.org/10.1099/mgen.0.000094>.

11 Benson G, (1999), Tandem Repeats Finder: a program to analyze DNA sequences, Nucleic Acids Research, <https://doi.org/10.1093/nar/27.2.573>.

12 Jolley KA, Maiden MCJ, (2010), BIGSdb: scalable analysis of bacterial genome variation at the population level, BMC Bioinformatics, <https://doi.org/10.1186/1471-2105-11-595>.

13 Edgar RC, (2004), MUSCLE: multiple sequence alignment with high accuracy and high throughput, Nucleic Acids Research, <https://doi.org/10.1093/nar/gkh340>.

14 Suyama M, Torrents D, Bork P, (2006), PAL2NAL: robust conversion of protein sequence alignments into the corresponding codon alignments, Nucleic Acids Research, <https://doi.org/10.1093/nar/gkl315>.

15 Korber B, (2000), HIV Signature and Sequence Variation Analysis. Computational Analysis of HIV Molecular Sequences., Kluwer Academic Publishersmmunity, <https://doi.org/10.1007/b112102>.

16 Karp PD, Billington R, Caspi R, et al., (2017), The BioCyc collection of microbial genomes and metabolic pathways, Briefings in Bioinformatics, <https://doi.org/10.1093/bib/bbx085>.

17 Wattam AR, Davis JJ, Assaf R, et al., (2017), Improvements to PATRIC, the all-bacterial bioinformatics database and analysis resource center, Nucleic Acids Research, <https://doi.org/10.1093/nar/gkw1017>.

18 Arndt D, Grant JR, Marcu A, et al., (2016), PHASTER: a better, faster version of the PHAST phage search tool, Nucleic Acids Research, <https://doi.org/10.1093/nar/gkw387>.

19 Bagos PG, Nikolaou EP, Liakopoulos TD, Tsirigos KD, (2010), Combined prediction of Tat and Sec signal peptides with hidden Markov models, Bioinformatics, <https://doi.org/10.1093/bioinformatics/btq530>.

20 Petersen TN, Brunak S, von Heijne G, Nielsen H, (2011), SignalP 4.0: discriminating signal peptides from transmembrane regions, Nature methods, <https://doi.org/10.1038/nmeth.1701>.

21 Krogh A, Larsson B, von Heijne G, Sonnhammer EL, (2001), Predicting transmembrane protein topology with a hidden Markov model: application to complete genomes, Journal of molecular biology, <https://doi.org/10.1006/jmbi.2000.4315>.

22 Wang Y, Huang H, Sun Ma, Zhang Q, Guo D, (2012), T3DB: an integrated database for bacterial type III secretion system, BMC Bioinformatics, <https://doi.org/10.1186/1471-2105-13-66>.

23 Fallarino A, Mavrangelos C, Stroeher UH, Manning PA, (1997), Identification of additional genes required for O-antigen biosynthesis in *Vibrio cholerae* O1, Journal of Bacteriology, <https://doi.org/10.1128/jb.179.7.2147-2153.1997>.

24 Bhumiratana A, Siriphap A, Khamsuwan N, Borthong J, Chonsin K, Sutheinkul O, (2014), O Serogroup-Specific Touchdown-Multiplex Polymerase Chain Reaction for Detection and Identification of *Vibrio cholerae* O1, O139, and Non-O1/Non-O139, Biochemistry research international, <https://doi.org/10.1155/2014/295421>.

25 Dorman MJ, Domman D, Uddin MI, et al., (2019), High quality reference genomes for toxigenic and non-toxigenic *Vibrio cholerae* serogroup O139, Sci Rep, <https://doi.org/10.1038/s41598-019-41883-x>.

26 Winter EE, Goodstadt L, Ponting CP, (2004), Elevated rates of protein secretion, evolution, and disease among tissue-specific genes, Genome Research, <https://doi.org/10.1101/gr.1924004>.

27 Nogueira T, Touchon M, Rocha EPC, (2012), Rapid Evolution of the Sequences and Gene Repertoires of Secreted Proteins in Bacteria, PLoS One, <https://doi.org/10.1371/journal.pone.0049403>.
